# PMLB: a large benchmark suite for machine learning evaluation and comparison

**DOI:** 10.1186/s13040-017-0154-4

**Published:** 2017-12-11

**Authors:** Randal S. Olson, William La Cava, Patryk Orzechowski, Ryan J. Urbanowicz, Jason H. Moore

**Affiliations:** 1 Institute for Biomedical Informatics, University of Pennsylvania, 3700 Hamilton Walk, Philadelphia, 19104 PA USA; 2 Department of Automatics and Biomedical Engineering, AGH University of Science and Technology, Kraków, Poland

**Keywords:** Machine learning, Model evaluation, Benchmarking, Data repository

## Abstract

**Background:**

The selection, development, or comparison of machine learning methods in data mining can be a difficult task based on the target problem and goals of a particular study. Numerous publicly available real-world and simulated benchmark datasets have emerged from different sources, but their organization and adoption as standards have been inconsistent. As such, selecting and curating specific benchmarks remains an unnecessary burden on machine learning practitioners and data scientists.

**Results:**

The present study introduces an accessible, curated, and developing public benchmark resource to facilitate identification of the strengths and weaknesses of different machine learning methodologies. We compare meta-features among the current set of benchmark datasets in this resource to characterize the diversity of available data. Finally, we apply a number of established machine learning methods to the entire benchmark suite and analyze how datasets and algorithms cluster in terms of performance. From this study, we find that existing benchmarks lack the diversity to properly benchmark machine learning algorithms, and there are several gaps in benchmarking problems that still need to be considered.

**Conclusions:**

This work represents another important step towards understanding the limitations of popular benchmarking suites and developing a resource that connects existing benchmarking standards to more diverse and efficient standards in the future.

## Introduction

The term *benchmarking* is used in machine learning (ML) to refer to the evaluation and comparison of ML methods regarding their ability to learn patterns in ‘benchmark’ datasets that have been applied as ‘standards’. Benchmarking could be thought of simply as a sanity check to confirm that a new method successfully runs as expected and can reliably find simple patterns that existing methods are known to identify [1]. A more rigorous way to view benchmarking is as an approach to identify the respective strengths and weaknesses of a given methodology in contrast with others [2]. Comparisons could be made over a range of evaluation metrics, e.g., power to detect signal, prediction accuracy, computational complexity, and model interpretability. This approach to benchmarking would be important for demonstrating new methodological abilities or simply to guide the selection of an appropriate ML method for a given problem.

Benchmark datasets typically take one of three forms. The first is accessible, well-studied *real-world data*, taken from different real-world problem domains of interest. The second is *simulated data*, or data that has been artificially generated, often to ‘look’ like real-world data, but with known, underlying patterns. For example, the GAMETES genetic-data simulation software generates epistatic patterns of association in ‘mock’ single nucleotide polymorphism (SNP) data [3, 4]. The third form is *toy data*, which we will define here as data that is also artificially generated with a known embedded pattern but without an emphasis on representing real-world data, e.g., the parity or multiplexer problems [5, 6]. It is worth noting that the term ‘toy dataset’ has often been used to describe a small and simple dataset such as the examples included with algorithm software.

While some benchmark repositories and datasets have emerged as more popular than others, ML still lacks a central, comprehensive, and concise set of benchmark datasets that accentuate the strengths and weaknesses of established ML methods. Individual studies often restrict their benchmarking efforts for various reasons, for example based on comparing variants of the ML algorithm of interest. The genetic programming (GP) community has also previously discussed appropriate benchmarking when comparing GP methodologies [7–9]. Benchmarking efforts may focus on a specific application of interest, e.g. traffic sign detection [10], or a more narrowly defined ML problem type, e.g. classification of 2-way epistatic interactions [11, 12]. The scope of benchmarking may also be limited by practical computational requirements.

There are currently a number of challenges that make it difficult to benchmark ML methods in a useful and globally accepted manner. For one, there are an overwhelming number of publications that reference the use of benchmark datasets, however there are surprisingly few publications that discuss the topic of appropriate ML benchmarking in general. Additionally, collecting and curating real-world benchmark datasets remains a challenge for many researchers [13]. Although repositories such as the UCI ML repository [14] and Kaggle [15] provide dozens of real-world datasets to download for free, these datasets come in myriad formats and require considerable preprocessing before ML methods can be applied to them. As a result, many benchmark datasets go unused simply because they are too difficult to preprocess. In addition, repositories such as Kaggle and OpenML [16] focus on solving data science problems through collaboration, and are not designed with comprehensive ML benchmarking in mind. Further, while real-world benchmarks can be derived from many different problem domains, from a strict data science perspective, many of the benchmarks in repositories can have very similar meta-features (e.g. the number of instances, number of features, number of classes, presence of missing data, and similar signal to noise ratios, etc.), such that while they are representative of different real-world problems, they may not represent a diverse assembly of data science problems. This issue has been raised previously: when applying UCI datasets as benchmarks, it was noted that the scope of included datasets limited method evaluation, and suggested that repositories such as UCI should be expanded [13, 17, 18].

Another challenge in benchmarking is that researchers often use only a handful of datasets when evaluating their methods, which can make it difficult to properly compare one ML method to the state-of-the-art ML methods [13]. For example, these datasets may be handpicked to highlight the strengths of the proposed method, while failing to demonstrate the proposed method’s potential weaknesses. As a result, although a ML method may perform well on a handful of datasets, it may fail to generalize to a broader range of problems. We submit that it is just as important to clearly identify the limitations of an algorithm in benchmarking practices, something that is often overlooked. While there will always be a need to identify and generate custom benchmarks for new or specialized problem domains, e.g. physical activity monitoring data [19] or dynamical systems simulation [20], it is vital for the bioinformatics and ML community to have a comprehensive benchmark suite with which to compare and contrast ML methods. Towards this goal, the present study introduces the Penn Machine Learning Benchmark (PMLB), a publicly available dataset suite (accessibly hosted on GitHub) initialized with 165 real-world, simulated, and toy benchmark datasets for evaluating supervised classification methods. PMLB includes datasets from many of the most-used ML benchmark suites, such as KEEL [21] and the UCI ML repository [14]. In addition to collecting data from these resources, PMLB standardizes the format of these data and provides useful interfaces for fetching datasets directly from the web.

This initial PMLB repository is not meant to be comprehensive; it includes mainly real-world datasets and excludes regression datasets (i.e. those with a continuous-valued dependent variable), as well as any datasets with missing values. We have chosen to focus our initial assessment on available datasets in classification. This paper includes a high-level analysis of the properties (i.e. meta-features) of the founding PMLB datasets, such as feature counts, class imbalance, etc. Further, we evaluate the performance of 13 standard statistical ML methods from scikit-learn [22] over the full set of PMLB datasets. We then assess the diversity of these benchmark datasets from the perspective of their meta-features as well as based on the predictive performance over the set of ML methods applied. Beyond introducing a new simplified resource for ML benchmarks, this study was designed to provide insight into the limitations of currently utilized benchmarks, and direct the expansion and curation of a future improved PMLB dataset suite that more efficiently and comprehensively allows for the comparison of ML methods. This work provides another important step toward the assembly of a effective and diverse set of benchmarking standards integrating real-world, simulated, and toy datasets for generalized ML evaluation and comparison.

## Penn machine learning benchmark (PMLB)

We compiled the Penn Machine Learning Benchmark (PMLB) datasets from a wide range of existing ML benchmark suites including the UCI ML repository [13, 14], Kaggle [15], KEEL [21], and the meta-learning benchmark [23]. As such, the PMLB includes most of the real-world benchmark datasets commonly used in ML benchmarking studies.

To make the PMLB easier to use, we preprocessed all of the datasets to follow a standard row-column format, where the features correspond to columns in the dataset and every instance in the data set is a row. All categorical features and labels with non-numerical encodings were replaced with numerical equivalents (e.g., “Low”, “Medium”, and “High” were replaced with 0, 1, and 2). Additionally, in every dataset, the dependent variable column was labeled as “class”. For multiclass datasets, we removed the instances of any class that had fewer than 10 instances for that class because < 10 instances are too few to reasonably learn on. Finally, all benchmark datasets with missing data were excluded from PMLB, as many ML algorithms cannot handle missing data in their standard implementations and we wished to avoid imposing a particular data imputation method in this initial study.

Currently, the PMLB consists of datasets for supervised classification (binary and multiclass). In supervised classification, we wish to find a mapping ${\hat {y}(\mathbf {x}): \mathbb {R}^{p} \rightarrow \mathcal {Y}}$ that associates the vector of features $\mathbf {x} \in \mathbb {R}^{p}$ with class labels from the set $\mathcal {Y} = \{1\;\dots \;K\}$ using *N* paired examples from the training set $\mathcal {T} = \{(\mathbf {x}_{i},y_{i}), i = 1\;\dots \;N\}$. In the future we plan to expand PMLB to include datasets for regression.

### PMLB meta-features

**Fig. 1:**
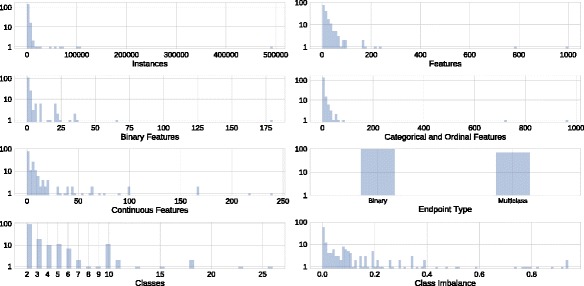
Histograms showing the distribution of meta-feature values from the PMLB datasets. Note the log scale of the y axes

In the current release, the PMLB includes 165 datasets. The meta-features of these datasets are summarized in Fig. 1. These meta-features are defined as follows:

- **# Instances**: The number of instances in each dataset.
- **# Features**: The number of features in each dataset.
- **# Binary Features**: The number of categorical features in each dataset with only two levels.
- **# Categorical and Ordinal Features**: The number of discrete features in each dataset with > 2 levels.
- **# Continuous Features**: The number of continuous-valued features in each dataset. Discriminating categorical and ordinal features from continuous features was determined automatically based on whether a variable was considered to be a ‘float’ in a Pandas DataFrame [24].
- **Endpoint Type**: Whether each dataset is a binary or multiclass supervised classification problem. Again, continuous endpoints for regression have been excluded in this study.
- **# Classes**: The number of classes to predict in each dataset’s endpoint.
- **Class Imbalance**: The level of class imbalance in each dataset ∈[0 1), where 0.0 corresponds to perfectly balanced classes and a value approaching 1.0 corresponds to extreme class imbalance, i.e. where nearly all instances have the same class value. Imbalance is calculated by measuring the squared distance of each class’s instance proportion from perfect balance in the dataset, as:
  $$I = K \sum_{i=1}^{K}{\left(\frac{n_{i}}{N} - \frac{1}{K}\right)^{2}} $$
  where *n*_*i*_ is the number of instances of class $i \in \mathcal {Y} $.

Most of the datasets have under 5000 instances and 500 features, and a fairly balanced class distribution. Roughly half of the datasets are binary classification problems, whereas the remaining half are multiclass classification problems ranging from 3-26 classes. Of the 165 datasets, 49 datasets have a mix of discrete (i.e. binary, categorical or ordinal) and continuous features, while 12 include only binary features, and 53 contain only continuous features. It is worth noting that the PMLB datasets cover a broad range of application areas, including biomedical studies, signal processing, and image classification, among others.

### PMLB Python interface

To make the PMLB datasets easier to access, we published an open source Python interface for PMLB on PyPi^1^. This interface provides a simple fetch_data function that returns any dataset in the PMLB as a *pandas* [24] DataFrame. For example, to fetch the clean2 dataset:



The clean2_data variable will then contain a data frame of the clean2 dataset, where the class column corresponds to the class labels and the remaining columns are the features. The fetch_data function has several caching and preprocessing options, all of which are documented in the PMLB repository^2^.

To acquire a full list of all datasets available in PMLB, users can access the dataset_names variable:



which is simply a Python list that contains the names of all PMLB datasets. For the remainder of the experiments described below, we used this Python interface to load the datasets prior to analysis.

## Evaluating machine learning methods

To provide a basis for comparison, we evaluated 13 supervised ML classification methods from scikit-learn [22] on the 165 datasets in PMLB. The methods and the parameters that were tuned are listed in Table 1. For more information on these ML methods, see [1] and the scikit-learn documentation [22].

**Table 1:** Machine learning algorithms and parameters tuned in the PMLB benchmark

Machine learning algorithm	Tuned parameters
Gaussian Naïve Bayes (NB)	No parameters.
Bernoulli Naïve Bayes	alpha: Additive smoothing parameter.
	binarize: Threshold for binarizing the features.
	fit_prior: Whether or not to learn class prior probabilities.
Multinomial Naïve Bayes	alpha: Additive smoothing parameter.
	fit_prior: Whether or not to learn class prior probabilities.
Logistic regression	C: Regularization strength.
	penalty: Whether to use Lasso or Ridge regularization.
	fit_intercept: Whether or not the intercept of the linear
	classifier should be computed.
Linear classifier trained via stochastic gradient	loss: Loss function to be optimized.
descent (SGD)	penalty: Whether to use Lasso, Ridge, or ElasticNet
	regularization.
	alpha: Regularization strength.
	learning_rate: Shrinks the contribution of each successive
	training update.
	fit_intercept: Whether or not the intercept of the linear
	classifier should be computed.
	l1_ratio: Ratio of Lasso vs. Ridge reguarlization to use.
	Only used when the ‘penalty’ is ElasticNet.
	eta0: Initial learning rate.
	power_t: Exponent for inverse scaling of the learning rate.
Linear classifier trained via the passive aggressive	loss: Loss function to be optimized.
algorithm	C: Maximum step size for regularization.
	fit_intercept: Whether or not the intercept of the linear
	classifier should be computed.
Support vector machine for classification (SVC)	kernel: ‘linear’, ‘poly’, ‘sigmoid’, or ‘rbf’.
	C: Penalty parameter for regularization.
	gamma: Kernel coef. for ‘rbf’, ‘poly’ & ‘sigmoid’ kernels.
	degree: Degree for the ‘poly’ kernel.
	coef0: Independent term in the ‘poly’ and ‘sigmoid’ kernels.
K-Nearest Neighbor (KNN)	n_neighbors: Number of neighbors to use.
	weights: Function to weight the neighbors’ votes.
Decision tree	min_weight_fraction_leaf: The minimum number of
	(weighted) samples for a node to be considered a leaf.
	Controls the depth and complexity of the decision tree.
	max_features: Number of features to consider when
	computing the best node split.
	criterion: Function used to measure the quality of a split.
Random forest & Extra random forest	n_estimators: Number of decision trees in the ensemble.
(a.k.a. Extra Trees Classifier)	min_weight_fraction_leaf: The minimum number of
	(weighted) samples for a node to be considered a leaf.
	Controls the depth and complexity of the decision trees.
	max_features: Number of features to consider when
	computing the best node split.
	criterion: Function used to measure the quality of a split.
AdaBoost	n_estimators: Number of decision trees in the ensemble.
	learning_rate: Shrinks the contribution of each successive
	decision tree in the ensemble.
Gradient tree boosting	n_estimators: Number of decision trees in the ensemble.
	learning_rate: Shrinks the contribution of each successive
	decision tree in the ensemble.
	loss: Loss function to be optimized via gradient boosting.
	max_depth: Maximum depth of the decision trees.
	Controls the complexity of the decision trees.
	max_features: Number of features to consider when
	computing the best node split.

We evaluated the ML methods using *balanced accuracy* [25, 26] as the scoring metric, which is a normalized version of accuracy that accounts for class imbalance by calculating accuracy on a per-class basis then averaging the per-class accuracies. When we evaluated each ML method, we first scaled the features of the datasets by subtracting the mean and scaling the features to unit variance. This scaling step was necessary for some ML methods, such as the K-Nearest Neighbor classifier, which assumes that the datasets will be scaled appropriately beforehand. (Note that the datasets provided in PMLB are not scaled nor normalized in order to keep them as close as possible to their original form.)

Once the datasets were scaled, we performed a comprehensive grid search of each of the ML method’s parameters using 10-fold cross-validation to find the best parameters (according to mean cross-validation balanced accuracy) for each ML method on each data set. This process resulted in a total of over 5.5 million evaluations of the 13 ML methods over the 165 data sets. For a comprehensive parameter search, we used expert knowledge about the ML methods to decide what parameters and parameter values to evaluate. The complete code for running the experiment is available online^3^. It should be noted that due to the different number of parameters for each algorithm, not every algorithm had the same number of evaluations.

## Results

In order to characterize the datasets in PMLB, they are clustered based on their meta-features in “Dataset meta-features” section. We then analyze the datasets based on ML performance in “Model-dataset biclustering” section, which identifies which datasets can be solved with high or low accuracy, as well as which datasets are appear universally easy or hard for the set of different ML algorithms to model accurately versus which ones appear to be particularly useful for highlighting differential ML algorithm performance.

### Dataset meta-features

**Fig. 2:**
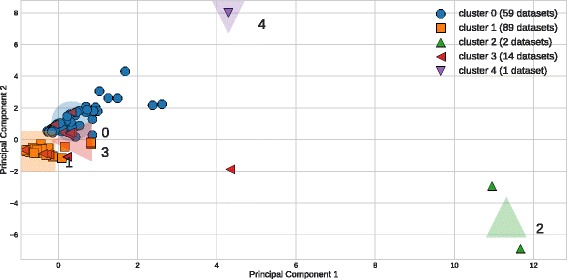
Clustered meta-features of datasets in the PMLB projected onto the first two principal component axes (PCA 1 and PCA 2)

**Fig. 3:**
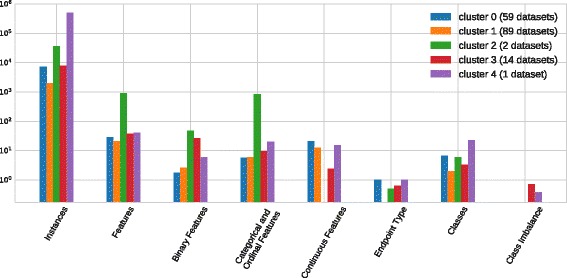
Mean values of each meta-feature within PMLB dataset clusters identified in Fig. 2

We used *k*-means to cluster the normalized meta-features of the datasets into 5 clusters, visualized along the first two principal component axes in Fig. 2 (note that the first two components of the PCA explain 49% of the variance, so we expect there to be some overlap of clusters in visualization). The number of clusters was chosen to compromise between the interpretability of the clusters and the adequate separation of the clustered datasets, as defined by the silhouette score. Figure 2 includes two clusters centered on outlier datasets (clusters 2 and 4). All clusters are compared in more detail according to the mean values of the dataset meta-features in each cluster in Fig. 3. Clusters 0 and 1 contain most of the datasets, and are separated by their endpoint type, i.e. cluster 0 is comprised of binary classification problems, whereas cluster 1 is comprised of multiclass classification problems. Cluster 2 is made up of 3 datasets with relatively high numbers features (a GAMETES dataset with 1000 features and the MNIST dataset with 784). Cluster 3 contains datasets with high imbalance between classes in the data set. Finally, cluster 4 is reserved for the KDD Cup dataset, which has exceptionally high number of instances (nearly 500,000). The clustering analysis thus reflects fairly intuitive ways in which the challenges presented by a particular dataset can be categorized, namely: large numbers of instances, large numbers of features, high class imbalance, and binary versus multiclass classification.

### Model-dataset biclustering

**Fig. 4:**
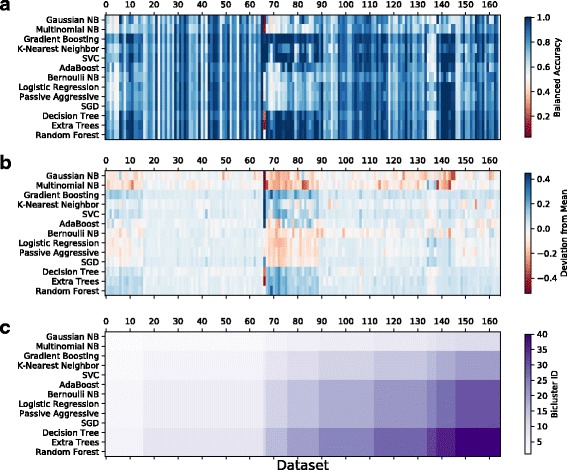
**a** Biclustering of the 13 ML models and 165 datasets according to the balanced accuracy of the models using their best parameter settings. **b** Deviation from the mean balanced accuracy across all 13 ML models. Highlights datasets upon which all ML methods performed similarly versus those where certain ML methods performed better or worse than others. **c** Identifies the boundaries of the 40 contiguous biclusters identified based on the 4 ML-wise clusters by the 10 data-wise clusters

Figure 4 summarizes the results of biclustering the balanced accuracy of the tuned models according to the ML method and dataset using a spectral biclustering algorithm [27]. The methods and datasets are grouped into 40 contiguous biclusters (4 ML-wise clusters by 10 data-wise clusters) in order to expose relationships between models and datasets. Figure 4 a presents the balanced accuracy. Figure 4 b preserves the clustering from ‘A’, but presents the deviation from the mean balanced accuracy among all 13 ML methods, in order to clearly identify datasets upon which all ML methods perform similarly, and those where some methods performed better than others. Figure 4 c simply delineates the 40 identified biclusters defined by balanced accuracy biclustering in Fig. 4 a and preserved in Fig. 4 b.

It is interesting to note that the ML methods tend to group according to their underlying approach; for example, Gaussian and Multinomial Naïve Bayes methods cluster together, Logistic Regression, Passive Aggressive and SGD cluster together (all hyperplane estimators), and the tree-based methods Decision Tree, Extra Trees and Random Forest also form a separate cluster. Datasets that are derived from the same origin are observed to cluster in certain instances. For example, dataset cluster 1 (i.e. the left-most dataset cluster identified in the Fig. 4 c, including 4 separate biclusters) contains most of the GAMETES data sets; cluster 2 contains most of the mfeats datasets and the Breast Cancer datasets; and cluster 10 includes both of the Wine Quality datasets and several thyroid-related datasets (new-thyroid, allhyper, allbp, allrep).

Figure 4 a allows us to interpret the utility of certain datasets in terms of difficulty across *all* methods and across *classes* of methods. For example, the light-blue stripes of low balanced accuracy indicate that none of the models achieve good performance on datasets 22, 118, and 164, which correspond to the GAMETES Epistasis datasets that are known to be difficult due to the lack of univariate correlations between features and classes and the high amount of noise. In contrast, nearly every method solves dataset 140 (clean2) with a high degree of accuracy because there are simple linear correlations between the features and classes and no noise.

Other clusters of datasets and ML methods reveal contrasts in performance. Dataset cluster 3 is the only cluster to contain a single dataset, the parity5 problem, corresponding to dataset 66 in Fig. 4. This is a unique problem in which a ML method must be able to quantify whether the *number* of features with a given binary value is even or odd in order to correctly classify each instance. As a result, methods that consider the main effect of features independently are not able to solve it (e.g. the Naïve Bayes methods). In contrast, methods with high capacity for interactions between features do well (e.g. Gradient Boosting, K-Nearest Neighbor, SVC). This contrast is also seen in cluster 4 (datasets 67 - 75), which contains several datasets with strong interactions between features (e.g. tic-tac-toe, parity5+5, and multiplexer-6). Again we observe a contrast between ML methods that make assumptions of linear independence and those that do not across this cluster of datasets. Contrasting Fig. 4 a with Fig. 4 b helps to differentiate differences in overall performance on given datasets from differences in performance based on selected ML methodology. One important observation is that a reasonably large proportion of benchmarks included in this study yielded similar performance over the spectrum of ML methods applied. This is likely because the signals identified in these datasets were either universally easy or difficult to detect. Furthermore, for those datasets where variable performance was observed, often a group of datasets clustered together with a similar signature of better than average/worse than average performance (see Fig. 4 b).

**Fig. 5:**
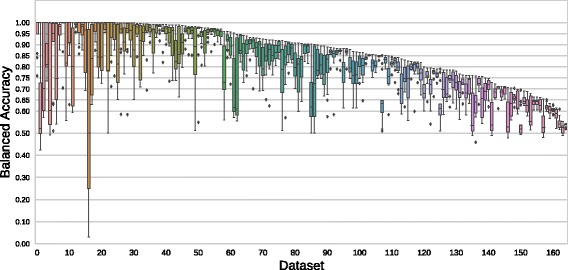
Accuracy of the tuned ML models on each dataset across the PMLB suite of problems, sorted by the maximum balanced accuracy obtained for that dataset

Overall, the current suite of datasets span a reasonable range of difficulty for the tested ML approaches. Figure 5 shows the distribution of scores for each tuned ML method for each dataset in the suite, sorted by best balanced accuracy score achieved by any method. The left-most dataset corresponds to clean2, mentioned above, and the right-most is analcatdata_dmft, with a maximum accuracy score of 0.544 for the methods tested. Approximately half (87) of the current suite can be classified with a balanced accuracy of 0.9 or higher, and nearly all (98.8%) of the datasets can be classified with a balanced accuracy of 0.6 or higher. Thus, although a range of model fidelity is observed, the datasets are biased towards problems that can be solved with a higher balanced accuracy.

## Discussion and conclusion

The primary goal of this paper is to introduce an ongoing research project for benchmarking ML methods. Specifically, we have collected and curated 165 datasets from the most popular data repositories and introduced PMLB, a new evolving set of benchmark standards for comparing and evaluating different ML methods. Apart from the repository itself, we have conducted a comprehensive analysis of the performance of numerous standard ML methods, which may be used as a baseline for evaluating and comparing newly developed ML methods. We also assessed the diversity of these existing benchmark datasets to identify shortcomings to be addressed by the subsequent addition of further benchmarks in a future release.

Simplicity and diversity are the ultimate priorities of the PMLB suite. This motivated us to clean and standardize the presentation of datasets in the repository, develop a simple interface for fetching data, and include datasets from multiple sources. Interestingly, when we analyzed the meta-features of the datasets in PMLB, we found that most of the datasets fall into a handful of categories based on feature types, class imbalance, dimensionality and numbers of classes. Notably, these findings align with recent studies suggesting that the UCI repository datasets lack the diversity to properly evaluate and compare ML methods [13, 18]. We also found that by biclustering the performance of a set of different ML algorithms on the datasets, we could observe classes of problems and algorithms that work well or poorly in conjunction.

Of course, PMLB is not yet a fully comprehensive benchmark suite for supervised classification methods. For instance, it currently excludes datasets with missing values or regression tasks and PMLB only has a handful of highly imbalanced datasets. One approach to adding diversity, pursued by the KEEL repository and related projects [13, 18, 21], is to augment existing benchmark repositories by injecting missingness, noise, and other relevant meta-features into existing datasets. However, in future work we propose to avoid adding multiple variants of the same dataset, and instead identify and simulate entirely new datasets with varying properties and meta-features to expand the PMLB suite and “fill in the gaps” of underrepresented problem types from a data science perspective. As in the present study, we plan to use performance comparisons over a diversity of ML methods in order to identify a limited set of benchmark standards able to diversely identify methodological advantages and disadvantages.

We expect this future work to lead to a more comprehensive benchmark tool that will better aid researchers in discovering the strengths and weaknesses of ML methods, and ultimately lead to more thorough—and honest—comparisons between ML methods.

## Footnotes

1
https://pypi.python.org/pypi/pmlb/

2
https://github.com/EpistasisLab/penn-ml-benchmarks

3
https://github.com/rhiever/sklearn-benchmarks/tree/master/model_code/
